# Changes in Circulating ProAMH and Total AMH during Healthy Pregnancy and Post-Partum: A Longitudinal Study

**DOI:** 10.1371/journal.pone.0162509

**Published:** 2016-09-09

**Authors:** Michael W. Pankhurst, Christine A. Clark, Judith Zarek, Carl A. Laskin, Ian S. McLennan

**Affiliations:** 1 Department of Anatomy, Otago School of Medical Sciences, University of Otago, Dunedin, New Zealand; 2 Lunenfeld-Tanenbaum Research Institute, Mount Sinai Hospital, Toronto, Canada; 3 LifeQuest Centre for Reproductive Medicine, Toronto, Canada; 4 Division of Clinical Pharmacology and Toxicology, Department of Pediatrics, Hospital for Sick Children, Toronto, Canada; Center For Human Reproduction, UNITED STATES

## Abstract

Circulating Anti-Müllerian hormone (AMH) is derived from the gonads, and is a mixture of the prohormone (proAMH), which does not bind to AMH receptors, and receptor-competent AMH. The functions of a hormone are partially defined by the factors that control its levels. Ovarian reserve accounts for 55~75% of the woman-to-woman variation in AMH level, leaving over 25% of the biological variation to be explained. Pregnancy has been reported to decrease circulating AMH levels, but the observations are inconsistent, with the effect of pregnancy on the bioactivity of AMH being unknown. We have therefore undertaken a longitudinal study of circulating proAMH and total AMH during pregnancy. Serum samples were drawn at 6–8 gestational time-points (first trimester to post-partum) from 25 healthy women with prior uneventful pregnancies. The total AMH and proAMH levels were measured at each time-point using ELISA. The level of circulating total AMH progressively decreased during pregnancy, in all women (p<0.001). On average, the percentage decline between the first trimester and 36–39 weeks’ gestation was 61.5%, with a standard deviation of 13.0% (range 30.4–81.2%). The percentage decline in total AMH levels associated with maternal age (R = -0.53, p = 0.024), but not with the women’s first trimester AMH level. The postpartum total AMH levels showed no consistent relationship to the woman’s first trimester values (range 31–273%). This raises the possibility that a fundamental determinant of circulating AMH levels is reset during pregnancy. The ratio of proAMH to total AMH levels exhibited little or no variation during pregnancy, indicating that the control of the cleavage/activation of AMH is distinct from the mechanisms that control the total level of AMH.

## Introduction

Anti-Müllerian hormone (AMH) is a member of the TGFβ superfamily, which are pleiotropic context-dependent regulators [1]. The AMH in the circulation is of gonadal origin [2], although paracrine production of AMH occurs in the uterus, mature brain and possibly other sites [3, 4]. TGFβ ligands are synthesised as proproteins, which are enzymatically cleaved at the site of synthesis. Some proproteins are bioactive [5, 6], although most are simply precursors, with bioactivity only occurring after cleavage. The cleavage of proAMH appears to be physiologically regulated as the extent of cleavage varies with the stage of development in a gender-specific manner, with additional variation occurring between similar individuals [7, 8]. In all instances, the cleavage of proAMH is inefficient, with circulating AMH being a mixture of both AMH_N,C_ and proAMH [7]. Commercial AMH ELISAs do not distinguish between proAMH and AMH_N,C_, and therefore provide an aggregate measure of two AMH species (Total AMH) [9]: one of which is bioactive on AMH receptors (AMH_N,C_), with the bioactivity of the other (proAMH) being unknown, beyond its inability to activate AMH receptors [10]. ProAMH is not cleaved by human serum *in vitro* [9], and no AMH_N,C_ accumulates in the circulation after intravenous injection of proAMH to mice [11]. This suggests that the extent of cleavage of circulating AMH is regulated by the state of the gonads [11].

AMH derives its name from its role in the regression of the uterine precursor in male embryos [12]. AMH has a broad role in the generation of the male phenotype [13], and immature males have uniquely high levels of AMH [14]. However, the AMH gene appears to have evolutionary-conserved roles in both sexes [13, 15]. In females, AMH modules the ovarian response to follicle stimulating hormone (FSH) [16], but it is unknown whether the underlying mechanism involves a local paracrine action of AMH or whether the ovarian functions of AMH are mediated by circulating AMH. AMH receptors are present in the uterus [3, 17], the breast [18] and other tissues, suggesting that circulating AMH may have physiological functions that are currently unknown.

The physiological functions of hormones are partly defined by the factors that determine their levels. Ovarian reserve is the major determinant of AMH levels in women, with AMH being extensively studied as a biomarker for this reason [19]. However, variation in antral follicle count only accounts for 55~75% of the woman-to-woman variation in AMH levels [20–22], indicating that other determinants of circulating AMH levels exists. Vitamin D status [23, 24] and the stage of the ovarian cycle [25] are determinants of circulating AMH levels, but their influence is minor. Pregnancy has been reported to decrease circulating AMH levels, but the observations are inconsistent and only include partial time courses [26–30]. We have therefore undertaken a longitudinal study to verify if and when AMH levels decline during pregnancy, and whether the cleavage state / bioactivity of maternal circulating AMH varies during gestation.

## Materials and Methods

### Participant selection

The study sample included 25 healthy women with a history of at least one uneventful term pregnancy prior to the index pregnancy, who had participated in another observational study of pregnancy reported elsewhere [31]. Participants were only selected for this study if they had at least 6 serum samples drawn throughout the index pregnancy and post-partum period. All pregnancies were uneventful and resulted in live-term babies. Demographic data, gestational outcome, and hematocrit measures in each trimester were collected by chart review. Fetal sex was not an experimental variable, but has been recorded in Table 1, as the relationship between maternal AMH levels and fetal sex is a matter of current interest [27, 32]. All participants provided written informed consent. The Institutional Review Board at Mount Sinai Hospital, Toronto, Canada approved the study. The study conforms to the principles expressed in the Declaration of Helsinki.

**Table 1:** Characteristics of the participants.

Age	Total AMH (pmol/L)			PP/1^st^ trimester (%)		Fetal sex	BF	pp
	1^st^ trimester	3^rd^ trimester	PP	AMH	Hematocrit			
Woman with no significant PP AMH rebound								
33	17.7	5.7	5.5	31	104.6	M	N	9
42	10.7	2.8	3.8	36	102.6	M	Y	14
37	38.2	14.0	18.9	49	112.2	F	N	7
37	5.5	2.1	2.8	51	112.0	M	Y	6
38	9.4	3.1	5.3	57	120.6	F	Y	6
Woman with a PP rebound to near 1^st^ trimester value								
29	2.3	1.6	2.2	98	92.7	F	Y	8
37	29.9	(9.0)	29.5	(99)	103.4	F	Y	12
32	60.5	(19.7)	59.7	(99)	114.6	M	Y	7
21	33.3	13.8	33.6	101	107.9	M	Y	5
43	13.3	2.5	14.1	106	102.7	M	Y	8
33	16.3	4.2	17.5	107	107.9	M	Y	12
38	19.2	9.7	22.4	117	108.4	F	Y	14
39	13.8	4.9	16.3	118	111.1	M	Y	6
30	11.2	7.1	13.2	118	112.6	M	Y	5
24	8.0	NA	9.5	119	113.8	M	N	28
34	22.1	(13.5)	27.3	(124)	119.4	F	Y	10
Woman with a PP rebound to well above 1^st^ trimester value								
35	20.9	5.5	31.2	150	105.4	M	Y	28
32	2.1	0.8	3.2	150	104.0	M	Y	8
35	6.6	2.1	11.2	169	105.8	F	Y	12
35	18.3	7.6	41.7	227	105.1	F	Y	7
36	10.9	3.7	26.1	239	NA	M	Y	12
26	32.2	13.4	88.1	273	108.4	M	Y	14

The women’s ages are in years. BF is whether the mother was breastfeeding when the post-partum sample was collected (Y = yes; N = no). PP is week’s post-partum. The sex of the fetuses are recorded as M for male and F for female.

### Sample collection and handling

Serum was collected monthly starting at the first prenatal visit following a positive βhCG test, between 11.5 and 12.5 weeks gestation, until 36–39 weeks gestation. Gestational timing and fetal heartbeat were confirmed by Doppler ultrasound at the first visit. Whenever possible, a final post-partum serum sample was collected. All samples were stored at -80°C until tested. Samples had been thawed and frozen twice: once during the previous study [31] and once to be aliquoted and shipped on dry ice from Toronto to Otago for AMH testing. AMH is stable through multiple freeze-thaw cycles [33, 34].

### AMH assay

Total AMH was measured in duplicate using the AMH Gen II assay (Beckman Coulter, Cat# A79765, following field safety notice FSN-20434-3, June 2013) according to the manufacturer’s specified protocol. ProAMH was measured according to a modified protocol of the Gen II assay, as previously described [35]. The AMH Gen II calibrators (Beckman Coulter, Cat# A79766) were used for quantification in total AMH immunoassays and a recombinant human proAMH standard was used for quantification in proAMH immunoassays [35]. A recombinant human AMHN,C negative control [35] was included in the ELISA run. All samples were measured in two batches on consecutive days, to minimise assay variation. The intra-assay % coefficients of variations were 5.4 and 5.2 for the total AMH and proAMH ELISAs, respectively. ELISA sample concentrations were calculated from standard curves fitting to quadratic equations (Prism 6, Graphpad Software).

### Statistical analysis and calculations

The AMH prohormone index (API) was calculated as the relative proportion of proAMH, expressed as a percentage of total AMH (API = [proAMH]/[total AMH] * 100). The percentage change in AMH values and the API during pregnancy was calculated using natural logs. Values in each woman’s series were normalised to her first trimester value. Three women lacked a first-trimester sample, and were excluded from this analysis. Samples were not available for 2 women at 16–19 weeks, 5 women at 24–27 weeks, 2 women at 32–35 and 4 women at 36–39 weeks. The influence of gestational week on AMH level was therefore assessed using a repeated measures mixed model. All women with a first-trimester sample had a post-partum sample. Regression analysis was used to determine whether maternal age, first trimester AMH or post-partum AMH level influenced the magnitude of the decline in AMH during pregnancy or the magnitude of the post-partum rebound. First trimester and post-partum AMH and hematocrit values were directly compared by a paired t-test. Statistical analyses were performed using either IBM SPSS Statistics or Sigma Plot 11.0 (Systat Systems, San Jose, CA). Power calculations were made using G*power, with alpha error probability set at 0.05 [36].

## Results

### Participants and samples

**Fig 1:**
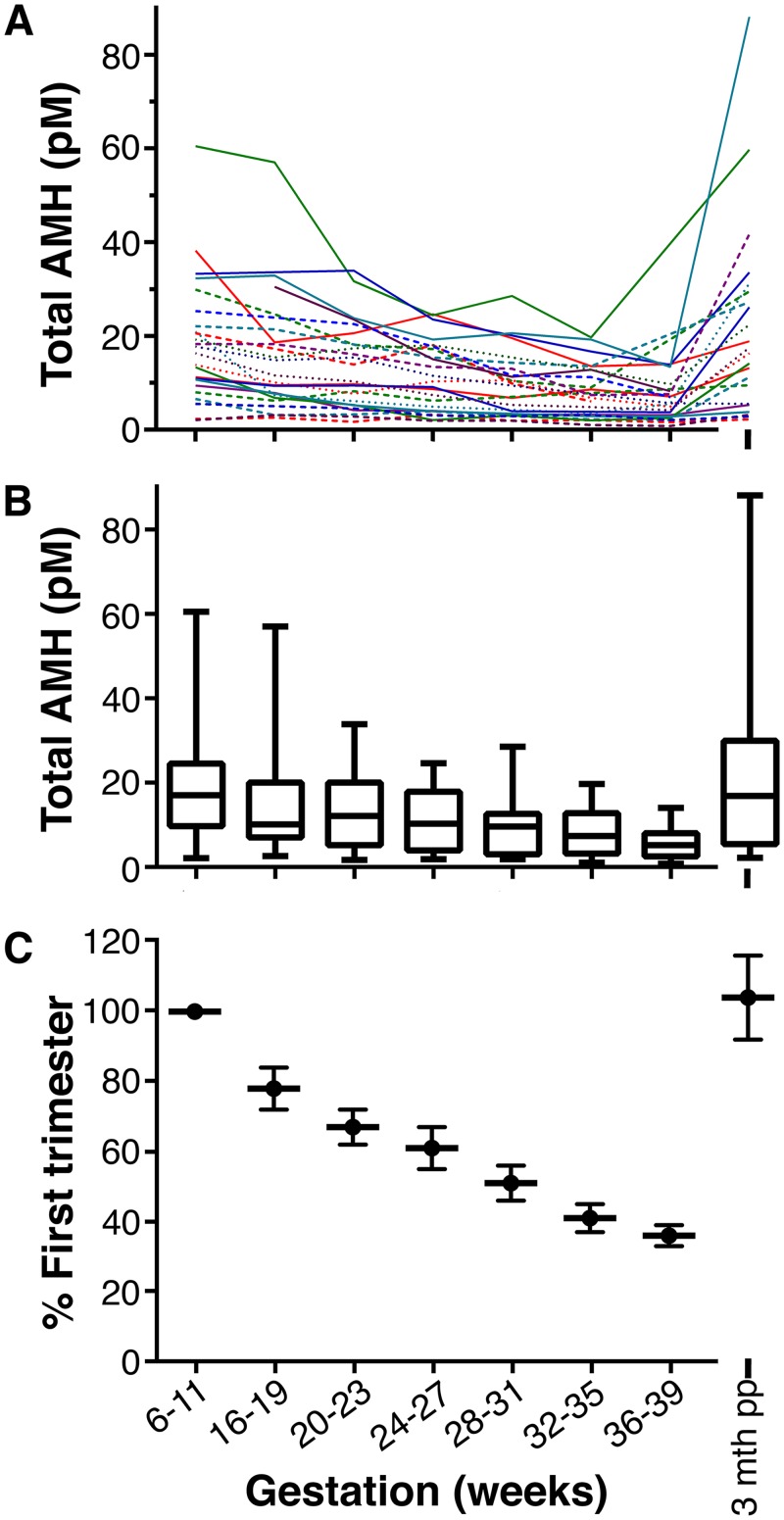
Change in total AMH levels during gestation. (A) Each woman’s individual levels are shown. (B) Box and whisker plots (medians, interquartile intervals, range) of AMH levels. (C) Percentage decline. Each woman’s values were normalized to her first trimester (6–11 weeks) sample, using log transformation. The data is the mean ± standard of error of the mean. There is a significant decline with gestational age, p<0.001 (repeated measures, mixed model). LSD post-hoc analysis indicated that all time points were significantly different compared to the 6–11 week samples (p = 0.026 for the 16–19 week time-point and p < 0.001 for the other gestational ages). 1 ng/ml of AMH = 7.14 pmol/L (pM).

The mean age of the women was 33.7 years (median: 34.5; range: 21–43). The 25 pregnancies resulted in term deliveries of 15 male and 10 female infants. Nineteen women were breastfeeding at the time of their post-partum sample. Three women lacked a first-trimester sample. Their data was included in the descriptive analysis (Fig 1A) but was excluded from the statistical analysis, as the first-trimester value serves as the normalising variable.

### Change in AMH during pregnancy

**Fig 2:**
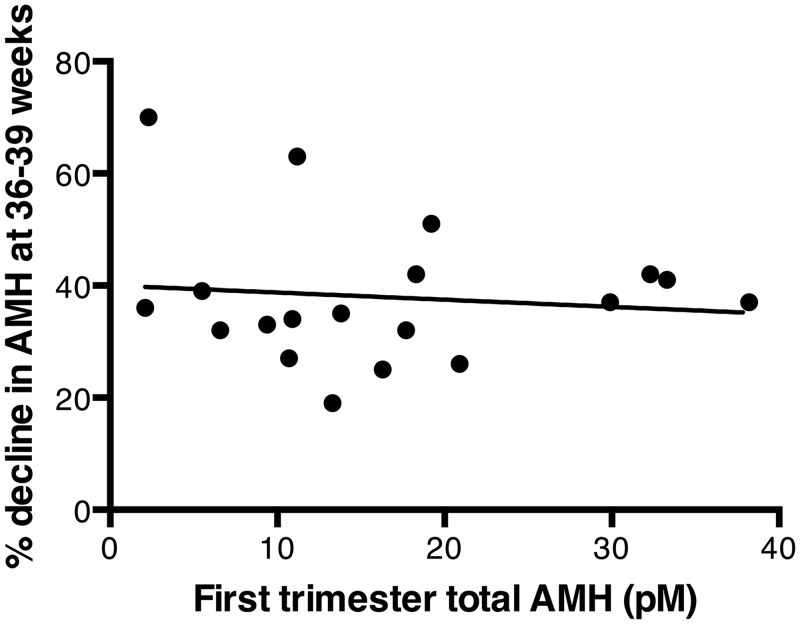
The magnitude of pregnancy-related decline was dissociated from a woman’s level of AMH. Each woman’s level of total AMH during the first trimester is plotted against the extent to which her total AMH levels declined by 36–39 weeks gestation. The two measures showed no significant correlation (R = - 0.04). 1 ng/ml of AMH = 7.14 pmol/L (pM).

**Fig 3:**
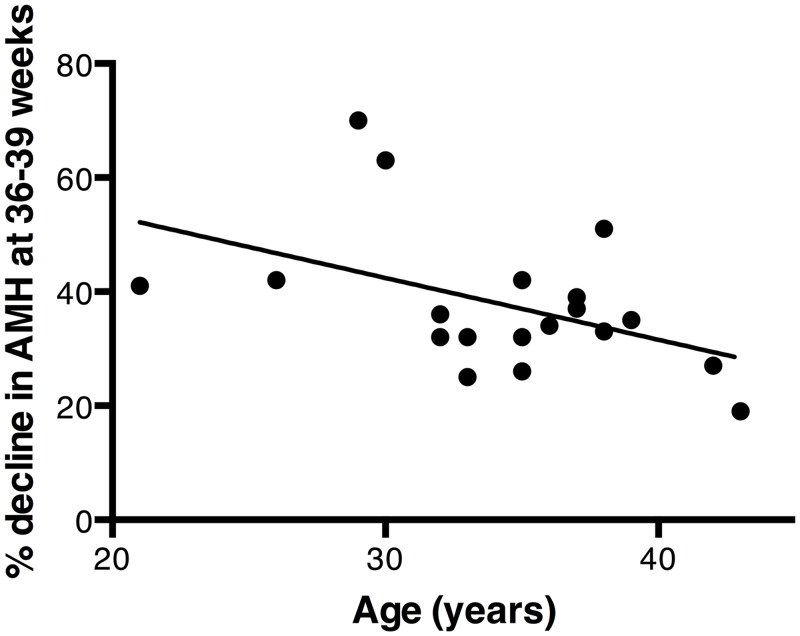
The influence of maternal age on the pregnancy-associated decline in total AMH level. The percentage decrease in total AMH levels between the first trimester and the 36–39 week samples is plotted against the women’s ages. The two parameters were significantly correlated, R = -0.53, p = 0.024.

All women exhibited a progressive decline in AMH during pregnancy with a mean decrease at 36–39 weeks’ gestation of 61.5% (SD: 13.0; range 30.4–81.2%) compared to first trimester AMH values (p<0.001, Fig 1). The magnitude of the pregnancy-associated loss in AMH values varied greatly between individuals, with this variation being dissociated from the woman’s first trimester AMH value (Fig 2). This suggests that ovarian reserve is not a determinant of the pregnancy-associated decrease in circulating AMH. However, there was a statistically significant trend for older women to exhibit a proportionally greater loss in AMH levels during pregnancy (R = -0.53, p = 0.024) (Fig 3). The combination of age and initial AMH were not stronger predictors of the decline in AMH in a multi-regression analysis than age alone: the standardised β for age in the model was -0.61 (p = 0.016), whereas the standardised β for first trimester AMH was -0.24 (NS, p = 0.31).

Pregnancy-related plasma volume increases have been proposed as a causal factor of AMH declines in pregnancy [37]. Declines in hematocrit are negatively correlated with plasma volume increases [38] hence we investigated whether declines in hematocrit were associated with declines in AMH. The mean decrease in hematocrit from the first to third trimesters was 7.6% (SD: 4.0%; range: 0–14.6%), but there was no correlation between the decrease in AMH and in hematocrit (r^2^ = 0.058).

### Post-partum AMH levels

The first trimester and post-partum values of most women (16/22) were different, to a level beyond assay variation (> 2 CV) (Table 1). Six women exhibited post-partum levels that were very high relative to all of their pregnancy values, whereas five women exhibited the opposite trend, with little or no rebound in AMH levels post-partum. The presence of two opposite trends results in there being little difference on average between the time points (18.3 vs 22.0 pmol/L, n = 22, p = NS). Consequently, this is a rare occurrence where comparison of mean values is misleading. The percentage change between third trimester and post-partum samples did not correlate with the women’s age, first trimester AMH values, the number of post-partum weeks, or the hematocrit rebound (Table 1).

### Form of circulating AMH

**Fig 4:**
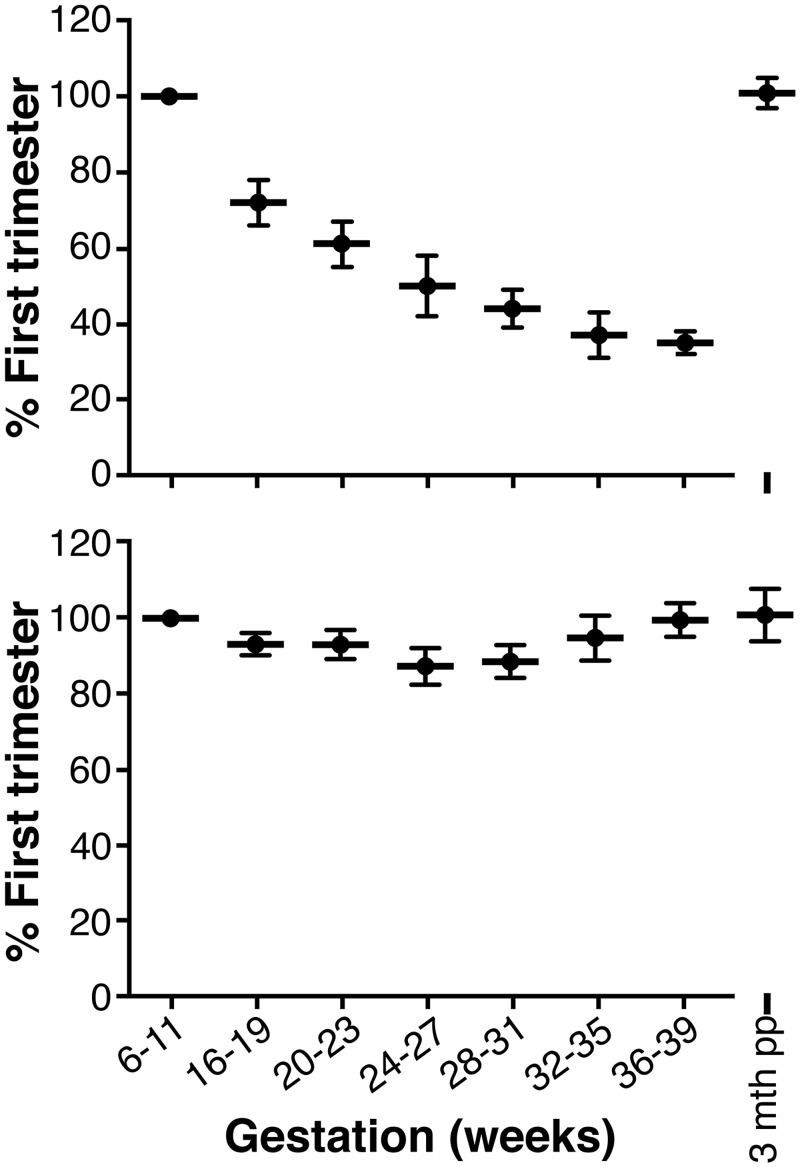
Change in the proportion of the AMH forms during gestation. (A) ProAMH levels during pregnancy. Each woman’s values were normalized to her first trimester (6–11 weeks) sample, using log transformation. The data is the mean ± standard of error of the mean. There is a significant decline with gestational age, p<0.001 (repeated measures, mixed model). LSD post-hoc analysis indicated that all gestational time points were significantly different compared to the 6–11 week samples (p < 0.001). (B) API. The ratio of proAMH to total AMH was calculated for each woman at each stage of pregnancy, and normalised to her first trimester (6–11 weeks) sample. The data is the mean ± standard of error of the mean. There was no significant effect of gestational age (repeated measures, mixed model).

The proAMH concentrations generally declined during gestation at an equivalent rate to AMH_N,C_, suggesting that pregnancy does not grossly influence the cleavage-activation of proAMH (Fig 4A). The extent of cleavage of proAMH is most accurately examined using the AMH prohormone index (API), which is the ratio of the proAMH and total AMH values [7]. The API was lower during weeks 24–31 than at other stages of pregnancy, but the magnitude of the difference was small (Fig 4B). When all time points were examined, the stage of gestation had no statistically significant effect on the API. The post-partum API values were not significantly different to the women’s first trimester values, and did not exhibit the large person-to-person variation that was present in the total AMH (Fig 4B).

## Discussion

The decline in circulating AMH during pregnancy was progressive, large (mean 62%), universal (25/25 women) and statistically robust (p<0.001). These findings confirm and extend previously longitudinal studies by Nelson et al [28] and Koninger et al [29]. The two reports of total AMH being invariant during pregnancy were cross-sectional [26, 27]. Cross-sectional studies of total AMH levels are statistically less powerful than longitudinal studies, due to the large inter-person variation in ovarian reserve. Power calculations from the current dataset indicate that group sizes of greater than 60 would be needed to detect a difference in total AMH levels between the first trimester and 36–39 week values, if a cross-sectional design was used.

### Postpartum AMH levels

The post-partum changes in circulating total AMH levels exhibited extreme variation between women. The existence of this phenomenon is not apparent when cohort means are examined, as the AMH levels of some woman rise whereas those of other women decline, creating a range of post-partum values that spanned 31% to 273% of the woman’s first trimester levels. AMH is a determinant of the rate of recruitment of primordial follicles [39], and this data may therefore indicate that the pattern of use of ovarian follicles is reset during pregnancy. The current study examined a single post-partum time point, as the existence of this phenomenon was not anticipated. Additional studies are needed to determine whether there is a sustained change in the woman’s AMH levels after pregnancy. If so, the determinants of the post-partum changes will need to be examined, as they may explain part of the variation in AMH levels which is unrelated to ovarian reserve. There was no apparent effect of breastfeeding or maternal age on the post-partum rebound in the current cohort, although this needs to be verified in studies that are specifically designed to test this. In particular, we suggest that there is a need to determine whether a change in post-partum AMH rebound correlates with a change in fecundity and/or is influenced by parity or gravidity. Until information such as this is known, we suggest that caution is needed when using AMH levels to measure ovarian reserve during pregnancy or after either an abortion or birth.

### ProAMH

In cross-sectional studies of healthy individuals, the API index does not correlate with total AMH levels, suggesting that the control of the activation of AMH is independent on the mechanisms that control the total level of AMH [7]. Similarly, in this study, the marked decline in total AMH levels during pregnancy was not matched by a concordant change in the cleavage-state of AMH, although a slight increase in the cleavage of circulating AMH was observed during the middle period of pregnancy. The extent of cleavage of AMH varies in other physiological circumstances [7, 40], with the regulation of this being currently unknown. The concentrations of ovarian steroid hormones change markedly during pregnancy [41]. If these hormones influence the cleavage of proAMH, then the API should vary during pregnancy, which was not the case. Hence, the cleavage of proAMH appears to be largely or totally independent of estrogens and progesterone.

The levels of total AMH fell markedly during pregnancy without a concordant increase in the API. Total AMH and the API exhibit minimal correlation in other physiological circumstances, indicating the amount of ovarian proAMH does not exceed the capacity of the enzymes to cleave it. ProAMH is putatively cleaved by furin, PCSK5 and plasmin. These enzymes have broad specificities and cleave multiple procytokines within the ovary (reviewed [13]). Consequently, the API is potentially a proxy for the activation of ovarian cytokines that are not released to the circulation.

### Mechanistic considerations

The pregnancy-related changes in AMH could arise through the accumulative effects of multiple physiological changes. La Marca et al [37] suggests that the increase in plasma volume during pregnancy leads to hemodilution of total AMH levels. This may be relevant to the initial decline in AMH levels, but it is not a complete explanation, as the increase in plasma volume is near complete by 25 weeks’ gestation [42–44], whereas total AMH levels continued to decline steadily from the 24–27 week time-point onwards. Large antral follicles are depleted during pregnancy, due to suppression of FSH-release, but this would only cause a minor decline in AMH levels as small antral follicle are retained during pregnancy [45–47]. The small antral follicles are the main producers of AMH in non-pregnant women [48], but this is not necessarily the case during pregnancy, as the physiological state of these follicles changes during pregnancy, as evidenced by an alteration in their morphology [45, 49]. In addition to the physiological state of the follicles, which may be hormonally regulated, there may be direct regulation of AMH production by reproductive hormones, such as estrogen, which putatively regulates AMH synthesis [50]. Levels of other reproductive hormones, such as LH, prolactin, activin and inhibin are also altered during pregnancy, each with differing rates of postpartum reversion to normal levels [41, 51–53]. The relationship between these hormones and AMH synthesis is not well characterised but the possibility that they are involved in regulating gravid AMH levels cannot be excluded.

The decline in circulating AMH during pregnancy begs the question of whether the decline in AMH has physiological consequences. AMH receptors are present in the uterus [3, 17], the placenta [54] and the breast [18], giving credence to this possibility, but experimental investigation is currently lacking. We emphasise, however, that successful pregnancy occurs across a wide range of AMH values, and that any pregnancy-related role for AMH would be to produce quantitative changes in physiological processes occurring in either the mother or her fetus.

### Limitation statement

This current study does not include a pre-pregnancy time point, and therefore does not necessarily show the full magnitude of the pregnancy-related change in circulating AMH. As noted above, the study is designed to detect the presence/absence of a change in circulating AMH, but is only able to provide indicative data regarding the influence of factors such as breastfeeding and maternal age.

### Fetal AMH

The sexes of the fetuses were recorded in this study, as several research groups have postulated an effect of fetal sex on maternal AMH levels [1–3]. This data has therefore been placed in the public domain, but no conclusions have been drawn for the reasons outlined below.

#### Size difference between the mother and fetus

The difference in the size of the mother and fetus limits the ability of a fetal blood protein to accumulate in the maternal circulation. When AMH is injected intravenously to mice, it rapidly disperses from the blood to the extracellular fluids [4], indicating that total maternal extracellular fluids need to be taken into account. During pregnancy, the average maternal extracellular fluid volume (V_m_) is approximately 15 L [5]. Fetal blood volume (V_f_) increases with gestational age rising from approximately 30 ml at 21 weeks gestation to approximately 190 mL by 35 weeks gestation [6]. The diluting effect of maternal fluids can be calculated from the formula V_m_.C_m_ = V_f_.C_f_; C_m_ = C_f_.(V_f_/V_m_), where C is concentration. At 21 weeks, the maternal concentration will be 30/15,000 (0.2%) of the fetal concentration rising to 1.3% at 35 weeks. The upper extreme of AMH in the fetal circulation of males is approximately 700 pmol/L, with most male fetuses have less than half of this level [1, 7–9]. At the upper extreme, the maternal concentration would rise by a maximum of 1.4 pmol/L at 21 weeks, increasing by a maximum of 9.1 pmol/L (1.3 ng/ml) near term. Female fetuses lack detectable levels of AMH during the first and second trimesters, with levels being below 15 pmol/L near term [1, 10]. Therefore, the upper limit for fetal AMH in the maternal circulation is less than 1 pmol/L. AMH levels range from 3–60 pmol/L for women in their twenties, failing to below 7 pmol/L in women older than 40 years [11].

#### Placental barrier

The above calculations indicate that if AMH in the maternal and fetal circulation were in equilibrium then AMH from a male fetus would have limited effect on maternal AMH levels, except when the male fetus is in the upper percentiles of AMH values and the mother’s AMH levels are in the lower percentiles. However, the placenta is a barrier to the free movement of proteins. Consequently, the level of fetal AMH in the maternal circulation will only rise above trace levels if AMH is actively transported from the fetal to the maternal circulation. To date, no theoretical rationale has been advanced for this existence of active transport of fetal AMH. The observational data produced to date is also insufficient to test whether fetal AMH is present in maternal circulation. AMH levels in women are highly variable [11], with the standard deviation for AMH in young women is greater than 20 pmol/L, which is large relative to the possible influence of fetal AMH. Hence, an extraordinarily large sample size will be need to prove/disprove the hypothesis that fetal AMH reaches the maternal circulation, unless both maternal and fetal AMH levels are known.

#### Pregnancy-related decline

The transport of fetal AMH to the maternal circulation could theoretically be detected by comparing pre-pregnancy and gestational blood samples, provided maternal AMH levels were constant during pregnancy. However, maternal AMH levels decline in all women during pregnancy, with the magnitude of the decline varying from woman-to-woman. This variation precludes using maternal AMH levels to predict the sex of a fetus. The above argument assumes that male fetuses do not regulate maternal AMH levels, and that placental-derived AMH [12] is not a significant determinant of circulating maternal AMH levels. The observation that maternal AMH levels decline during pregnancy is consistent with this.

## Conclusions

In conclusion, pregnancy leads to a change in total AMH level, without a concordant change in the activation of AMH. The magnitude of this phenomenon was large compared to all other known determinants of AMH levels, with the exception of the primary determinant, ovarian reserve. It is currently uncertain whether AMH levels return to a woman’s pre-pregnancy level during the post-partum period, creating significant uncertainty about the interpretation of total AMH as a biomarker of ovarian reserve. The physiological mechanism underlying the pregnancy-related decline in AMH is uncertain.
